# Vitamin D Status Determines Cardiometabolic Effects of Cabergoline in Women with Elevated Prolactin Levels: A Pilot Study

**DOI:** 10.3390/nu15102303

**Published:** 2023-05-14

**Authors:** Robert Krysiak, Marcin Basiak, Grzegorz Machnik, Witold Szkróbka, Bogusław Okopień

**Affiliations:** Department of Internal Medicine and Clinical Pharmacology, Medical University of Silesia, 40-752 Katowice, Poland

**Keywords:** cardiometabolic risk, dopamine agonists, hyperprolactinemia, vitamin D

## Abstract

Both hyperprolactinemia and vitamin D deficiency appear to be associated with increased cardiometabolic risk. This study aimed to determine whether vitamin D status influences the cardiometabolic effects of cabergoline. The study included three matched groups of women with mild to moderate hyperprolactinemia: vitamin D-naive subjects with vitamin D insufficiency (group A), women with vitamin D deficiency/insufficiency successfully treated with vitamin D (group B), and vitamin D-naive individuals with normal vitamin D status (group C). Plasma prolactin, 25-hydroxyvitamin D, estradiol, glucose homeostasis markers, lipids, high-sensitivity C-reactive protein (hsCRP), fibrinogen, homocysteine, and uric acid, as well as the urinary albumin-to-creatinine ratio (UACR), were measured at study entry and after four months of cabergoline treatment. Although cabergoline reduced prolactin levels and increased estradiol levels in all study groups, the effect on prolactin was more pronounced in groups B and C compared to group A. In groups B and C, the drug enhanced glucose homeostasis, increased HDL-cholesterol, and decreased triglycerides, hsCRP, fibrinogen, homocysteine, uric acid, and UACR. In group A, only insulin resistance, hsCRP, and homocysteine were reduced by cabergoline. The effects on insulin sensitivity, HDL-cholesterol, triglycerides, hsCRP, fibrinogen, homocysteine, uric acid, and UACR were proportional to the decrease in prolactin and baseline levels of 25-hydroxyvitamin D. The obtained results suggest that vitamin D status determines cabergoline’s cardiometabolic effects.

## 1. Introduction

The clinical consequences of hyperprolactinemia go beyond its typical symptoms, such as oligomenorrhea/amenorrhea, galactorrhea, impaired fertility and multidimensional impairment of sexual functioning. Prolactin excess results in hyperphagia, increased food intake and weight gain [1,2]. Consequently, long-term hyperprolactinemia promotes obesity/overweight, insulin resistance, impaired glucose tolerance and atherogenic dyslipidemia [3,4]. High levels of this hormone in patients with untreated prolactinoma were have been found to cause endothelial dysfunction and subclinical atherosclerosis, the advancement of which was dependent on the degree of prolactin hypersecretion [5]. Macrophages near the lipid core and shoulder regions of atherosclerotic plaques express numerous prolactin receptors [6]. Cardiometabolic risk factors, including high-sensitivity C-reactive protein (hsCRP), homocysteine, and uric acid, are significantly elevated in hyperprolactinemic patients compared to those with prolactin levels within the reference range [7,8]. Prolactin excess caused by prolactin-secreting tumors or other pituitary disorders increases procoagulant activity and inhibited fibrinolysis [8,9]. In a population-based study (Study of Health in Pomerania), circulating levels of this hormone were positively associated with mortality from all causes and cardiovascular disease [10]. Patients with acute coronary syndromes had a higher prolactin concentration than those with stable angina pectoris [11]. Hyperprolactinemia has also been identified as a risk factor for stroke, and this association was attributed to the chronic prolactin excess-induced increase in platelet reactivity [12]. Lastly, hyperprolactinemia impairs the cardiometabolic properties of lipid-lowering drugs [8,13]. Beyond normalizing prolactin levels, dopamine agonists, considered the first-line treatment for hyperprolactinemia [14], decreased plasma glucose, increased peripheral tissue insulin responsiveness, improved lipid profile, as well as improved anthropometric parameters (the body mass index (BMI), waist circumference and visceral fat content) [1,15,16,17,18,19]. Moreover, these drugs produced a systemic anti-inflammatory effect [7,20], increased flow-mediated dilation [7], and decreased thickness of the intima–media complex [21]. Although high-dose cabergoline treatment may increase risk of cardiac valve regurgitation [22], small doses of this agent (used in the treatment of prolactin excess) seem to exert more pronounced cardiometabolic effects than bromocriptine does [20].

Low vitamin D status may also play an adverse role in the development of insulin resistance, atherosclerosis, and their associated complications. Vitamin D (calciferol) deficiency are associated with increased values of systolic pressure, diastolic pressure, insulin levels, HOMA1-IR and triglycerides, as well as with decreased HDL-cholesterol concentrations in adolescents [23]. Low vitamin D levels are associated with endothelial dysfunction, ultrasonographic signs of subclinical atherosclerosis (increased carotid intima-media thickness, arterial wall stiffening, and an increased number of plaques in peripheral arteries), and left ventricular hypertrophy [24]. Increased prevalence of calciferol deficiency and low levels of 25-hydroxyvitamin D are associated with the majority of insulin resistance disorders, including obesity, type 2 diabetes, metabolic syndrome, and polycystic ovary syndrome [25]. Vitamin D deficiency ois associated with coronary heart disease and all-cause mortality in large cohort studies [26]. Moreover, it has been reported that 25-hydroxyvitamin D levels correlate with the severity of ischemic heart disease [27]. Animal studies suggest that the active form of vitamin D (calcitriol) binds to vitamin D, resulting in a variety of potential cardiovascular benefits, including reduced production of renin, relaxation of vascular smooth muscle cells, and a decrease in the production of atherosclerotic foam cells [28]. In clinical studies, vitamin D supplementation has been found to be beneficial for the treatment of metabolic syndrome-related complications, including insulin resistance, hyperglycemia, dyslipidemia, obesity, and hypertension [29].

Concomitant prolactin excess and low vitamin D status may increase cardiometabolic risk to a greater extent than the presence of either condition alone. However, prolactin and vitamin D interactions are poorly understood. Metformin has been found to reduce prolactin levels only if 25-hydroxyvitamin D levels were within the reference range, and this effect correlated with 25-hydroxyvitamin D levels [30]. However, metformin’s inhibitory effect on lactotrope secretory function was clearly inferior to that of dopaminergic agents, and the study focused on metformin’s prolactin-lowering properties [31]. Nevertheless, vitamin D deficiency in men diminishes the cardiometabolic effects of statins [32], the drugs that reduce cardiovascular morbidity and mortality [33]. Therefore, the purpose of this study was to determine whether vitamin D status influences the cardiometabolic effects of cabergoline.

## 2. Materials and Methods

The institutional committee on human research approved the study protocol, ensuring that it adhered to the Declaration of Helsinki’s ethical principles. All participants provided written informed consent after receiving verbal and written information about the study. As the study was non-randomized and all groups received the same drug, it did not meet the criteria for a clinical trial and was not required to be registered in a public database. The paper was prepared in accordance with the Enhancing the Quality and Transparency of Health Research (EQUATOR) Network guidelines for observational studies (STROBE).

### 2.1. Patients

**Figure 1:**
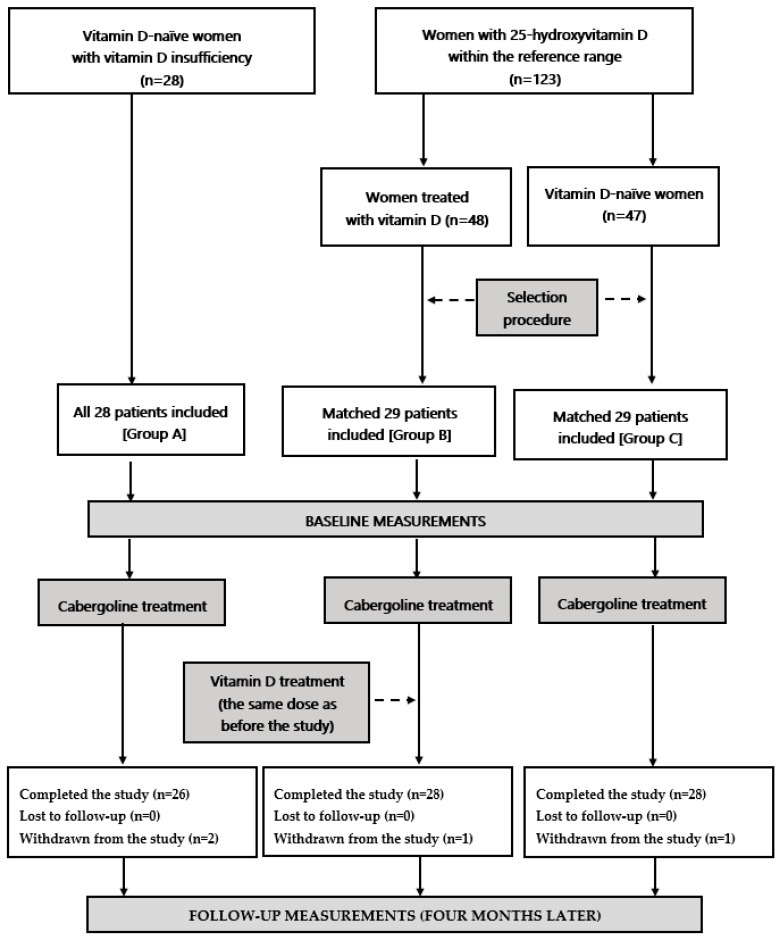
A diagram presenting the flow of patients in the study.

This prospective, non-randomized pilot study recruited women of reproductive age (18–45 years old) with mild or moderate hyperprolactinemia, defined as plasma total prolactin levels on two separate days between 30 and 80 ng/mL (640 and 1700 mU/L). Hyperprolactinemia was caused by microprolactinoma, antipsychotic drug treatment, traumatic brain injury, empty sella syndrome, or was idiopathic. Women who were either sexually inactive women or who were using non-hormonal contraception participated in the study. On the basis of plasma 25-hydroxyvitamin D levels measured on two separate occasions, the participants were assigned to one of three groups: vitamin D-naive women with vitamin D insufficiency (group A; *n* = 28); subjects with normal vitamin D status treated for at least 6 months with 50–100 μg (2000–4000 IU daily) of oral vitamin D preparations due to vitamin D deficiency/insufficiency (group B; *n* = 29); and vitamin D-naive women with normal vitamin D status (group C, *n* = 29). Vitamin D deficiency was defined as plasma 25-hydroxyvitamin D levels below 50 nmol/L (20 ng/mL), vitamin D insufficiency as plasma 25-hydroxyvitamin D levels between 50 and 75 nmol/L (20 and 30 ng/mL), and normal vitamin D status as plasma 25-hydroxyvitamin D levels between 75 and 150 nmol/L (30 and 60 ng/mL) [34]. To match study groups for age, body mass index (BMI), homeostatic model assessment 1 insulin resistance ratio (HOMA1-IR), and plasma prolactin levels, groups B and C were chosen from a larger pool of eligible participants (48 and 47, respectively). Group A consisted of all patients who met the inclusion criteria. Our study was adequately powered because an *a priori* power calculation indicated that 24 patients per group were required to detect a 20% between-group difference in the measured cardiometabolic risk factors. In order to restrict the influence of seasonal confounds and seasonal fluctuations in circulating levels of 25-hydroxyvitamin D and the remaining variables, similar numbers of individuals were recruited in each season: spring (22 women: 8 in group A, 7 in group B and 7 in group C), summer (22 women: 7 in group A, 8 in group B and 7 in group C), autumn (21 women: 7 in group A, 7 in group B and 7 in group C), and winter (21 women: 6 in group A, 7 in group B and 8 in group C). Figure 1 illustrates the flow chart of the patients throughout the study.

The exclusion criteria included macroprolactinomas, pituitary tumors co-secreting prolactin and other pituitary hormones, and pseudoprolactinomas, as severe hyperprolactinemia and pituitary tumors require specific treatment. For ethical reasons, we excluded vitamin D-naive women with vitamin D levels below 50 nmol/L (20 ng/mL). The remaining exclusion criteria were macroprolactinemia, other endocrine disorders, cardiovascular disease (except grade 1 hypertension), autoimmune or inflammatory diseases, renal or hepatic failure, malabsorption syndromes, any other serious disorders, premature or early menopause, pregnancy, or lactation, any pharmacotherapy (except antipsychotic drugs and exogenous vitamin D), and poor patient compliance.

### 2.2. Study Design

Throughout the entire study period (four months), all study groups received cabergoline, and group B received the same daily vitamin D dose as before the study. The starting dose of cabergoline increased from 0.25 mg once weekly in the first two weeks to 0.25 mg twice weekly beginning in the third week. In order to reduce the risk of pharmacokinetic interactions, vitamin D was administered in the morning, while cabergoline was administered at bedtime. The use of new prescription or over-the-counter medications was permitted if the treatment lasted less than seven days and was discontinued at least six weeks prior to completing the study. In addition, the patients were advised to adopt a healthier lifestyle (total fat intake <30% of total energy intake, saturated fat intake <7% of energy consumed, cholesterol intake <200 mg per day, fiber intake ≥15 g per 1000 kcal, and moderate to vigorous exercise for at least 30 min per day). The participants were evaluated every four weeks to ensure adherence to cabergoline treatment and improve study protocol compliance. Dietary counseling was provided by a certified nutritionist cooperating with our research team. During each visit, medication adherence was deemed satisfactory if the percentage of tablets returned fell between 0% and 10% and if all four questions in the Polish version of the Morisky–Green test were answered correctly. The daily vitamin D intake from food was determined by analyzing individual dietary questionnaires.

### 2.3. Laboratory Assays

All laboratory tests were conducted at the start of the study and again four months later. Each patient’s venous blood was drawn by trained phlebotomists in the follicular phase between 7:00 and 8:00 a.m., following a minimum 12 h overnight fast. All patients rested in a seated position for a minimum of 30 min prior to venipuncture. Experiments were carried out in duplicate by technicians without knowledge of the study protocol or participant characteristics. Plasma concentrations of glucose, lipids (total cholesterol, low-density lipoprotein (LDL)-cholesterol, high-density lipoprotein (HDL)-cholesterol and triglycerides), and uric acid, as well as urine concentrations of albumin and creatinine, were measured with commercial kits and standard methods (Roche Diagnostics, Basel, Switzerland). Glycated hemoglobin was measured in whole blood samples using a Cobas Integra 800 turbidimetric inhibition immunoassay (Roche Diagnostics, Mannheim, Germany). Insulin, prolactin, 25-hydroxyvitamin D, estradiol and homocysteine levels in plasma were measured using acridinium ester-based direct chemiluminescence (ADVIA Centaur XP Immunoassay System, Siemens Healthcare Diagnostics, Munich, Germany). Prolactin was measured both before (total prolactin) and after (monomeric prolactin) precipitation with polyethylene glycol, as previously described [35,36]. Plasma hsCRP levels were measured using an immunoassay with chemiluminescent detection (Immulite 2000XPi, Siemens Healthcare, Warsaw, Poland), while fibrinogen levels were determined using an automated BCS XP analyzer and the method of Clauss (Siemens Healthcare, Warsaw, Poland). HOMA1-IR was computed by multiplying plasma glucose (mg/dL) by plasma insulin (mU/L) and dividing the result by 405. Macroprolactin concentrations was calculated by subtracting monomeric prolactin concentrations from total prolactin concentrations.

### 2.4. Statistical Analysis

Due to skewed distributions, a natural log transformation was applied to all data. Baseline values, follow-up values, and percentage changes from baseline were compared using one-way analysis of variance followed by the *post hoc* Bonferroni test, after taking into account reasons for prolactin excess and sample collection season as potential confounders. Student’s paired *t*-test was used to conduct within-group comparisons. The χ2 test was used to assess nominal data. Correlations between the investigated variables were assessed using Pearson’s r tests for two continuous variables; phi coefficient for one continuous and one categorical variable; and point-biserial for two categorical variables. To find whether prolactin and vitamin D status have a significant effect on the measured cardiometabolic risk factors in the whole population, multivariate linear regression analysis with correction for age, cause of prolactin excess and BMI was carried out with cardiometabolic risk factors as dependent variables, and prolactin and 25-hydroxyvitamin D as independent variables. Possible relationships between vitamin D status and the impact of treatment on prolactin levels in the whole study population were verified by linear regression analysis (corrected for age, reason for hyperprolactinemia and BMI) with 25-hydroxyvitamin D levels as an independent variable. The data were analyzed using a predetermined significance level of *p*-value adjusted for multiple testing less than or equal to 0.05.

## 3. Results

Four patients were withdrawn from the study. One subject (assigned to group B) complained of dizziness and faintness when getting up from a sitting position. In another subject (assigned to group C), risperidone, an antipsychotic drug increasing prolactin levels, was replaced by a consultant psychiatrist with quetiapine. Lastly, two patients from group A required chronic treatment with other drugs (a non-steroidal anti-inflammatory agent and rosuvastatin). The remaining 82 patients (95%) completed the study, and their results were statistically analyzed. The analyzed women did not experience adverse effects associated with cabergoline and vitamin D treatment.

On the basis of the primary outcome data and the given sample size, a post hoc power calculation revealed that the study had sufficient statistical power (0.85). All patients analyzed complied with treatment and dietary recommendations. The average vitamin D intake from food did not differ between the study groups (group A: 10.8 ± 4.5 µg (432 ± 180 IU), group B: 11.2 ± 4.9 µg (448 ± 195 IU), group C: 11.5 ± 3.8 µg (11.5 ± 3.8 IU)). Physical activity and the average daily intake of calories, total fat, saturated fat, and cholesterol did not differ between study groups. In group B, the average daily dose of exogenous vitamin D was 76.8 ± 23.1 µg (3071 ± 923 IU), and the average duration of calciferol supplementation was 11 ± 3 months.

Age, smoking habits, causes of prolactin excess, percentage of patients with oligomenorrhea, BMI, waist circumference, blood pressure, prolactin (both monomeric and total), macroprolactin, estradiol, glucose, HOMA1-IR, glycated hemoglobin, lipids, hsCRP, fibrinogen, homocysteine, uric acid, and UACR did not differ significantly between study groups at baseline. However, hsCRP levels tended to be higher in group A than in groups B (*p* = 0.0724) and C (*p* = 0.0615). As expected, 25-hydroxyvitamin D levels were lower in group A than in the remaining two groups but were similar in groups B and C (Table 1 and Table 2).

In all study groups, cabergoline decreased total and monomeric prolactin levels, and increased estradiol levels. In groups B and C, cabergoline also decreased HOMA1-IR, glycated hemoglobin, triglycerides, hsCRP, fibrinogen, homocysteine, uric acid and UACR, as well as increasing HDL-cholesterol. In group A, the drug reduced HOMA1-IR, hsCRP and homocysteine. In neither group did cabergoline affect macroprolactin, 25-hydroxyvitamin D, glucose, total cholesterol or LDL-cholesterol. At follow-up, group A differed from the remaining groups in total prolactin, monomeric prolactin, 25-hydroxyvitamin D, HOMA1-IR, HDL-cholesterol, triglycerides, hsCRP, fibrinogen, homocysteine, uric acid and UACR (Table 2). There were no differences between baseline and follow-up smoking habits, BMI, waist circumference and blood pressure. Groups B and C differed from group A in the percentage changes from baseline in prolactin (total and monomeric), HOMA1-IR, HDL-cholesterol, triglycerides, hsCRP, fibrinogen, homocysteine, uric acid and UACR (Table 3).

In the whole cohort, baseline values of HOMA1-IR, glycated hemoglobin, triglycerides, hsCRP, fibrinogen, homocysteine, uric acid and UACR positively correlated with prolactin (r values between 0.28 (*p* = 0.0482) and 0.50 (*p* < 0.0001) for total prolactin, and between 0.34 (*p* = 0.0204) and 0.55 (*p* < 0.0001) for monomeric prolactin), and inversely correlated with 25-hydroxyvitamin D (r values between −0.31 (*p* = 0.0326) and −0.46 (*p* = 0.0002)). The opposite relationships were observed for HDL-cholesterol (total prolactin: r = −0.34; *p* = 0.0211; monomeric prolactin: r = −0.39; *p* = 0.0004; 25-hydroxyvitamin D: r = 0.41; *p* = 0.0006). Among all assessed risk factors, the strongest correlations with 25-hydroxyvitamin D were for hsCRP. Moreover, baseline estradiol correlated with baseline total (r = 0.38; *p* = 0.0012) and monomeric (r = 0.40; *p* = 0.0008) prolactin. Multivariate regression analysis showed that prolactin and 25-hydroxyvitamin D were independent and significant determinants of cardiometabolic risk factors (Table 4).

Cabergoline-induced reduction in prolactin levels positively correlated with 25-hydroxyvitamin D levels (r values between 0.38 (*p* = 0.0026) and 0.47 (*p* = 0.0001) for total prolactin, and between 0.40 (*p* = 0.0008) and 0.51 (*p* < 0.0001) for monomeric prolactin, depending on the group), and with treatment-induced increase in estradiol (r values between 0.37 (*p* = 0.0032) and 0.42 (*p* = 0.0006) for total prolactin, and between 0.39 (*p* = 0.0011) and 0.46 (*p* = 0.0002) for monomeric prolactin, depending on the group). Moreover, the decrease in prolactin levels positively correlated with the impact of treatment on HOMA1-IR, glycated hemoglobin, HDL-cholesterol, triglycerides, hsCRP, fibrinogen, homocysteine, uric acid and UACR (group A: r values between 0.28 (*p* = 0.0447) and 0.38 (*p* = 0.0012) for total prolactin, and between 0.30 (*p* = 0.0358) and 0.44 (*p* = 0.0004) for monomeric prolactin; group B: r values between 0.30 (*p* = 0.0385) and 0.48 (*p* = 0.0001) for total prolactin, and between 0.31 (*p* = 0.0295) and 0.52 (*p* < 0.0001) for monomeric prolactin; group C: r values between 0.29 (*p* = 0.0406) and 0.46 (*p* = 0.0002) for total prolactin, and between 0.31 (*p* = 0.0295) and 0.55 (*p* < 0.0001) for monomeric prolactin). The decrease in hsCRP positively correlated with both baseline 25-hydroxyvitamin D levels (group A: r = 0.43 (*p* = 0.0007); group B: r = 0.39 (*p* = 0.0015); group C: r = 0.37 (*p* = 0.0022)) and with the impact of treatment on HOMA1-IR, glycated hemoglobin, HDL-cholesterol, triglycerides, hsCRP, fibrinogen, homocysteine, uric acid and UACR (group A: r values between 0.30 (*p* = 0.0395) and 0.42 (*p* = 0.0008); group B: r values between 0.30 (*p* = 0.0371) and 0.41 (*p* = 0.0007); group C: r values between 0.29 (*p* = 0.0407) and 0.38 (*p* = 0.0014)). There were no correlations between the effect on the outcome variables and the daily dose of exogenous vitamin D contained in its preparations, duration of calciferol supplementation and dietary vitamin D intake. In linear regression analysis, treatment-induced changes in prolactin showed a relationship with 25-hydroxyvitamin D levels (Δ total prolactin: β = 0.370, adjusted R^2^ = 0.422, *p* < 0.0001; Δ monomeric prolactin: β = 0.402, adjusted R^2^ = 0.422, *p* < 0.0001).

## 4. Discussion

Although observed in all study groups, the decrease in prolactin levels was less pronounced in women with concomitant vitamin D deficiency, and follow-up levels of this hormone differed between patients with low vitamin D status and women with normal vitamin D status. This distinction cannot be explained by the characteristics of the study population at the outset. Because of a selection procedure, all groups were matched for age, BMI, insulin sensitivity and prolactin levels. In addition, there were no proportional differences between the groups for the various causes of prolactin excess. Due to the stringent inclusion and exclusion criteria, the impact of comorbidities or concurrent therapies cannot further explain these findings. Based on our observations, the only explanation for differences in plasma prolactin impact are between-group differences in vitamin D status. In accordance with this explanation, there was a correlation between the decrease in prolactin concentration and 25-hydroxyvitamin D levels. Moreover, in linear regression analysis, changes in prolactin showed a relationship with 25-hydroxyvitamin D levels. The results do not appear to be due to seasonal variation in 25-hydroxyvitamin D concentrations [37], as similar proportions of patients were recruited in each season, and sample collection season was considered a potential confounder in statistical analyses.

Irrespective of the study group, the decrease in total prolactin reflected a reduction in monomeric prolactin levels, while concentrations of macroprolactin, which is composed of high-molecular-weight complexes of prolactin and immunoglobulins (mainly immunoglobulin G) [38], did not change throughout the study. This finding is worth emphasizing for at least for two reasons. Firstly, although calciferol-induced increase in 25-hydroxyvitamin D levels was accompanied by a decrease in macroprolactin content in women with macroprolactinemia [35], differences in cabergoline action in the current study were not associated with interactions of cabergoline and calciferol at the level of high-molecular-weight prolactin. Secondly, even if macroprolactinemia increases cardiometabolic risk, this risk is much lower than in individuals with elevated levels of monomeric prolactin [36]. Thus, our findings are clinically relevant because they show changes in the hormone fraction with full biological activity.

Our study demonstrates for the first time that vitamin D status determines cabergoline’s cardiometabolic effects. In subjects with 25-hydroxyvitamin levels between 75 and 150 nmol/L, the drug improved insulin sensitivity, affected plasma lipids (increasing HDL-cholesterol and decreasing triglycerides), and decreased circulating levels of the remaining cardiometabolic risk factors: hsCRP, fibrinogen, homocysteine, uric acid, and UACR. In females with 25-hydroxyvitamin D levels between 50 and 75 nmol/L, the effect of this drug was limited to a reduction in HOMA1-IR, hsCRP, and homocysteine, which was less pronounced than in women with normal vitamin D status. This observation enables us to conclude that even slight disruptions in vitamin D homeostasis may reduce the effect of cabergoline on cardiometabolic risk factors. We can only hypothesize about the effect of 25-hydroxyvitamin D levels below 50 nmol/L on cabergoline action because patients with extremely low 25-hydroxyvitamin D levels require calciferol supplementation [39]. Nevertheless, correlations between 25-hydroxyvitamin D concentration and changes in all assessed risk factors suggest that cardiometabolic effects of cabergoline may be more perturbed or even absent in females with severe vitamin D deficiency compared to subjects with vitamin D insufficiency. 

An additional observation of interest was the absence of differences in the effects of cabergoline between two groups of women with prolactin levels within the reference range: vitamin D-naive women and women receiving exogenous calciferol due to a history of hypovitaminosis D. This finding suggests that the potent effects of cabergoline in vitamin D-treated hyperprolactinemic women reflect normal vitamin D status, and cannot be explained by the supplement’s effect. In accordance with this explanation, the prolactin-lowering and extra-prolactin effects of cabergoline did not correlate with the daily dose of exogenous vitamin D, the duration of calciferol supplementation, or vitamin D intake from food. Therefore, hypovitaminosis D does not appear to impair cardiometabolic effects of cabergoline if adequate amounts of exogenous calciferol are administered. Our findings also suggest that 25-hydroxyvitamin D levels should be measured routinely in hyperprolactinemic women at high risk for cardiovascular disease or diabetes, but ideally in all women with prolactin excess who require treatment with a dopamine agonist.

On the basis of the obtained results, certain inferences can be made. First, correlations between cardiometabolic risk factors and both prolactin and 25-hydroxyvitamin D levels suggest that hyperprolactinemia and low vitamin D status may make women more susceptible to cardiovascular and metabolic complications later in life, despite their young age. This risk may be especially high in individuals with severe hyperprolactinemia and severe hypovitaminosis D, who were excluded from our study for ethical reasons. Secondly, between-group differences in lowering prolactin levels suggest that cabergoline-resistant hyperprolactinemic patients should be evaluated for vitamin D deficiency/insufficiency, and if subnormal concentrations are detected, exogenous calciferol should be supplemented. In the event that 25-hydroxyvitamin D levels are low, this supplement may permit a reduction in cabergoline dosage. Thirdly, the benefits of cabergoline treatment likely extend beyond the reduction in prolactin levels and the resolution of prolactin-excess clinical symptoms. Cabergoline may prevent the onset of cardiovascular disease, type 2 diabetes, and other insulin-resistant conditions. This means that in hyperprolactinemic women in reproductive age, supplementation of exogenous calciferol is aimed not only at preventing osteomalacia, osteoporosis and bone fracture. Moreover, the presence of correlations between cabergoline action and 25-hydroxyvitamin D levels only in women with vitamin D deficiency suggests that the primary goal of calciferol supplementation in hyperprolactinemic women is to achieve 25-hydroxyvitamin D levels within the reference range, and that there is no specific target concentration for 25-hydroxyvitamin D associated with the strongest cabergoline effect. If prolactin excess is not complicated by irreversible changes in the vascular system and/or glucose homeostasis, calciferol supplementation-induced normalization of vitamin D status may reduce the likelihood of developing atherosclerosis, diabetes, and metabolic syndrome to the level observed in the general population of young women.

The obtained results do not support previous observations of our research team that cabergoline increases 25-hydroxyvitamin D in women with prolactin-secreting microadenoma treated ineffectively with bromoctiptine [20], nor do they support the findings of other authors [40], who reported lower 25-hydroxyvitamin D concentration in cabergoline-treated women with prolactinoma than in untreated females with prolactin levels within the reference range. Despite a relatively small sample size, the current study is the largest to evaluate the interaction between vitamin D status and excess prolactin. A larger sample size, assessment of not only total but also monomeric prolactin, and a small (25–31%) proportion of individuals with prolactin-secreting tumors (only microprolactinomas) may account for the inconsistency with previous findings. Intriguingly, despite the fact that female patients with prolactin-secreting tumors had significantly lower 25-hydroxyvitamin D concentrations than control subjects, 25-hydroxyvitamin D concentration was a predictor of prolactinoma size but not of prolactin levels [41]. Thus, it appears that differences between groups in cabergoline’s cardiometabolic effects cannot be attributed to this agent’s effect on vitamin D homeostasis.

Regarding the mechanisms explaining differences in cabergoline action, we can only speculate. Greater cardiometabolic effects in women with normal vitamin D homeostasis may be a direct result of differences in the degree of prolactin reduction between the groups. In accordance with this explanation, the reduction in all cardiometabolic risk factors correlated with the decrease in total and monomeric prolactin. Prolactin and calcitriol, the active form of vitamin D, may interact at the level of target tissue, regulating production of cardiometabolic risk factors. The opposite effect of both hormones on glucose transporter isoform 4, the primary insulin-responsive glucose transporter [42], seems to support this explanation. It was discovered that both a dopamine agonist [43] and calcitriol [44] upregulate the expression of this transporter, thereby increasing glucose uptake into fat and muscle cells. In individuals with vitamin D deficiency or insufficiency, this balance may be shifted, explaining only a moderate effect on levels of the assessed cardiometabolic risk factors. The results also provide indirect evidence linking the weak cardiometabolic effects of cabergoline with the systemic inflammation observed in subjects with low vitamin D status [45]. Although the difference did not reach statistical significance, hyperprolactinemic women with a coexisting vitamin D deficiency tended to have higher baseline hsCRP levels. In addition, correlation between hsCRP and 25-hydroxyvitamin D levels was stronger than correlations between 25-hydroxyvitamin D levels and the other risk factors. Lastly, the treatment-induced decrease in hsCRP positively correlated with both 25-hydroxyvitamin D levels and the remaining outcome measures. Rheumatoid arthritis, a systemic inflammatory disorder [46], is characterized by downregulation of D_2_ receptors [47]. Thus, systemic inflammation may counteract the effects of cabergoline by inhibiting the expression of D_2_ receptors. Our findings cannot be explained by changes in estradiol levels, reflecting a decrease in prolactin production. Although cabergoline increased estradiol concentration, which was relatively low at entry (because of prolactin excess), there were no between-group differences in estradiol levels at baseline and at follow-up, and treatment-induced changes in estradiol levels did not correlate with cardiometabolic effects of cabergoline.

There are several flaws that limit the validity of the conclusions. The current study was limited to a single center and recruited a small number of participants. Our findings should therefore be regarded as hypothesis-generating rather than conclusive. Latent confounding factors and selection bias may have had an impact on the results because the study was non-randomized and, for ethical reasons, did not include a group of women receiving placebo treatment. It is unknown whether vitamin D status influences the cardiometabolic effects of cabergoline in postmenopausal women and in men, not participating in the study. The study population included women with prolactin excess of various origin. It cannot be excluded that the impact of vitamin D status on cardiometabolic effects of cabergoline may depend on the reason of hyperprolactinemia. The study design does not make it possible to establish whether cardiometabolic effects were only a consequence of cabergoline action and vitamin D status because the participants received diet counselling during the study. The cardiometabolic risk factors evaluated in this study are merely surrogates for clinically relevant hard endpoints, such as morbidity and mortality. Because the study does not shed light on the cellular and molecular aspects of prolactin and calciferol interactions, the mechanisms underlying our findings must be elucidated. Lastly, despite the fact that the study protocol minimized the impact of random diurnal, seasonal, and analytical variations in the parameters measured, it was impossible to completely eliminate the regression toward the mean effect [48].

## 5. Conclusions

Although cabergoline decreased total and monomeric prolactin levels in all study groups, this effect was more pronounced in patients with 25-hydroxyvitamin D levels within the reference range than in women with levels below the reference range. The differences between the groups in lowering plasma prolactin were paralleled by differences in the impact on all assessed risk factors, with subjects with normal vitamin D status experiencing a greater impact. Cabergoline’s cardiometabolic effects correlated with its prolactin-lowering properties and 25-hydroxyvitamin D levels. Our findings suggest that low vitamin D status may impair cardiometabolic effects of cabergoline in women with mild to moderate prolactin excess, and that effective calciferol supplementation may prevent this unfavorable effect. Due to the pilot nature of the current study, its findings should be confirmed in longitudinal observational studies with sufficient sample sizes.

## Figures and Tables

**Table 1:** Baseline characteristics of patients.

Variable	Group A	Group B	Group C	*p*-Value		
				A vs. B	A vs. C	B vs. C
**Number** (n)	26	28	28	-	-	-
**Age** (years)	32 ± 7	31 ± 7	32 ± 6	0.6021	1.0000	0.5628
**Smokers** (%)/**Number of cigarettes a day** (n)/**Duration of smoking** (months)	35/8 ± 5/125 ± 48	32/9 ± 5/131 ± 46	36/8 ± 4/120 ± 42	0.8081	0.7656	0.4046
**Reasons for prolactin excess** (%): **microprolactinoma/drug-induced hyperprolactinemia/traumatic brain injury/empty sella syndrome/idiopathic**	31/38/15/8/8	29/39/14/11/7	25/36/18/11/11	0.7875	0.8015	0.8268
**Oligomenorrhea** (%) ^a,b^	77	71	75	0.3245	0.7405	0.5240
**BMI** (kg/m^2^)	24.0 ± 5.2	23.8 ± 5.0	23.6 ± 4.8	0.8860	0.7698	0.8792
**Waist circumference** (cm)	84 ± 9	83 ± 8	83 ± 7	0.6674	0.6492	1.0000
**Systolic blood pressure** (mmHg)	132 ± 14	130 ± 15	129 ± 16	0.6124	0.4641	0.8103
**Diastolic blood pressure** (mmHg)	86 ± 7	85 ± 7	84 ± 8	0.6021	0.3296	0.6207

Group A: Vitamin D-naive hyperprolactinemic women with vitamin D insufficiency; Group B: Hyperprolactinemic women with normal vitamin D status treated with exogenous vitamin D preparations because of vitamin D deficiency/insufficiency; Group C: Vitamin D-naive hyperprolactinemic women with normal vitamin D status. Unless otherwise stated, the data are presented as the mean ± standard deviation; ^a ^Oligomenorrhea was defined as menstrual interval greater than 37 days and less than 90 days. ^b^ There were no cases of amenorrhea (absence of menses for any period greater than 90 days) or polymenorrhea (menstrual interval less than 90 days). Abbreviations: BMI—body mass index.

**Table 2:** The effect of cabergoline on the investigated variables in women with prolactin excess.

Variable	Group A	Group B	Group C
**Total prolactin** (ng/mL)			
*Baseline*	55.1 ± 12.1	56.7 ± 12.5	57.3 ± 11.8
*Follow-up*	19.1 ± 6.7 *^#$^	11.6 ± 5.0 ^$^	12.0 ± 5.6 ^$^
**Monomeric prolactin** (ng/mL)			
*Baseline*	52.0 ± 11.7	53.2 ± 12.1	53.9 ± 11.4
*Follow-up*	16.3 ± 5.6 *^#$^	8.5 ± 4.0 ^$^	8.8 ± 4.2 ^$^
**Macroprolactin** (ng/mL)			
*Baseline*	3.1 ± 1.2	3.5 ± 1.7	3.4 ± 1.4
*Follow-up*	2.8 ± 0.9	3.1 ± 1.5	3.2 ± 1.2
**25-hydroxyvitamin D** (nmol/L)			
*Baseline*	61.8 ± 5.8 *^#^	107.2 ± 18.5	112.4 ± 17.2
*Follow-up*	62.4 ± 6.0 *^#^	111.2 ± 17.5	114.0 ± 16.5
** Estradiol ** (pmol/L)			
*Baseline*	125 ± 40	134 ± 42	129 ± 37
*Follow-up*	203 ± 62 ^$^	229 ± 70 ^$^	220 ± 68 ^$^
**Glucose** (mg/dL)			
*Baseline*	93 ± 14	91 ± 11	92 ± 12
*Follow-up*	90 ± 10	86 ± 11	86 ± 12
**HOMA1-IR**			
*Baseline*	3.5 ± 0.9	3.4 ± 1.0	3.5 ± 0.8
*Follow-up*	3.0 ± 0.8 *^#$^	2.2 ± 0.7 ^$^	2.1 ± 0.8 ^$^
**Glycated hemoglobin** (%)			
*Baseline*	5.2 ± 0.3	5.3 ± 0.4	5.3 ± 0.3
*Follow-up*	5.1 ± 0.2	5.0 ± 0.3 ^$^	5.1 ± 0.2 ^$^
**Total cholesterol** (mg/dL)			
*Baseline*	198 ± 32	202 ± 42	205 ± 39
*Follow-up*	196 ± 28	200 ± 37	198 ± 26
**HDL-cholesterol** (mg/dL)			
*Baseline*	50 ± 8	52 ± 10	49 ± 10
*Follow-up*	52 ± 8 *^#^	62 ± 11 ^$^	59 ± 10 ^$^
**LDL-cholesterol** (mg/dL)			
*Baseline*	116 ± 28	120 ± 34	123 ± 32
*Follow-up*	114 ± 30	114 ± 24	112 ± 26
**Triglycerides** (mg/dL)			
*Baseline*	144 ± 46	139 ± 36	147 ± 42
*Follow-up*	136 ± 40 *^#^	112 ± 27 ^$^	115 ± 30 ^$^
**hsCRP** (mg/L)			
*Baselin*	3.5 ± 1.1	3.0 ± 0.9	2.9 ± 1.2
*Follow-up*	2.8 ± 0.8 *^#$^	1.4 ± 0.7 ^$^	1.2 ± 0.6 ^$^
**Fibrinogen** (mg/dL)			
*Baseline*	375 ± 82	385 ± 90	400 ± 105
*Follow-up*	360 ± 92 *^#^	312 ± 86 ^$^	308 ± 82 ^$^
**Homocysteine** (μmol/L)			
*Baseline*	30.7 ± 11.6	28.5 ± 10.6	32.4 ± 14.0
*Follow-up*	22.1 ± 8.1 *^#$^	11.8 ± 5.5 ^$^	10.4 ± 4.3 ^$^
**Uric acid** (mg/dL)			
*Baseline*	5.0 ± 1.2	5.2 ± 2.0	4.8 ± 1.8
*Follow-up*	4.6 ± 1.4 *^#^	3.4 ± 1.0 ^$^	3.1 ± 1.2 ^$^
**UACR** (mg/g)			
*Baseline*	34.0 ± 11.5	36.8 ± 13.4	32.9 ± 12.5
*Follow-up*	30.6 ± 12.3 *^#^	12.5 ± 8.9 ^$^	13.2 ± 7.1 ^$^

Group A: Vitamin D-naive hyperprolactinemic women with vitamin D insufficiency; Group B: Hyperprolactinemic women with normal vitamin D status treated with exogenous vitamin D preparations because of vitamin D deficiency/insufficiency; Group C: Vitamin D-naive hyperprolactinemic women with normal vitamin D status; The data are presented as the mean ± standard deviation; * *p* < 0.05 vs. group B; ^#^ *p* < 0.05 vs. group C; ^$^ *p* < 0.05 vs. baseline value. Reference values for young women: total prolactin: 5.0–29.0 ng/mL; monomeric prolactin: 4.0–26.0 ng/mL; macroprolactin: 2.0–4.0 ng/mL; 25-hydroxyvitamin D: 75–150 nmol/L; estradiol: 80–550 pmol/L; glucose: 70–99 mg/dL, HOMA1-IR: <2.0; glycated hemoglobin: <5.6%; total cholesterol: <200 mg/dL; HDL-cholesterol ≥50 mg/dL; LDL-cholesterol: <115 mg/dL; triglycerides: <150 mg/dL; uric acid: 3.5–8.5 mg/dL; hsCRP: <1.0 mg/L; fibrinogen: 200–400 mg/dL; homocysteine: 4–14 μmol/L; UACR: <30 mg/g. Abbreviations: HDL—high-density lipoprotein; HOMA1-IR—the homeostatic model assessment 1 of insulin resistance ratio; hsCRP—high-sensitivity C-reactive protein; LDL—low-density lipoprotein; UACR—urinary albumin-to-creatinine ratio.

**Table 3:** Percentage changes from baseline in the investigated variables in hyperprolactinemic women receiving cabergoline.

Variable	Group A	Group B	Group C
** Δ ** **Total prolactin**	−65 ± 12	−80 ± 8 *	−79 ± 8 *
** Δ ** **Monomeric prolactin**	−69 ± 10	−84 ± 7 *	−84 ± 6 *
** Δ ** **Macroprolactin**	−10 ± 8	−11 ± 8	−6 ± 7
**Δ** **25-hydroxyvitamin D**	1 ± 5	4 ± 8	1 ± 4
** Δ Estradiol **	62 ± 28	71 ± 26	70 ± 24
** Δ ** **Glucose**	−3 ± 8	−5 ± 8	−7 ± 10
** Δ ** **HOMA1-IR**	−14 ± 15	−35 ± 20 *	−40 ± 22 *
** Δ ** **Glycated hemoglobin**	−2 ± 6	−6 ± 10	−4 ± 6
** Δ ** **Total cholesterol**	−1 ± 10	−1 ± 12	−3 ± 16
** Δ ** **HDL-cholesterol**	4 ± 8	19 ± 10 *	20 ± 12 *
** Δ ** **LDL-cholesterol**	−2 ± 10	−5 ± 11	−9 ± 18
** Δ ** **Triglycerides**	−8 ± 12	−19 ± 20 *	−22 ± 18 *
** Δ ** **hsCRP**	−20 ± 15	−53 ± 18 *	−59 ± 22 *
** Δ ** **Fibrinogen**	−4 ± 11	−19 ± 15 *	−23 ± 16 *
** Δ ** **Homocysteine**	−28 ± 20	−59 ± 18 *	−68 ± 16 *
** Δ ** **Uric acid**	−8 ± 15	−35 ± 15 *	−35 ± 17 *
** Δ ** **UACR**	−10 ± 20	−66 ± 15 *	−60 ± 18 *
** Δ BMI **	−1 ± 2	−2 ± 2	−2 ± 3

Group A: Vitamin D-naive hyperprolactinemic women with vitamin D insufficiency; Group B: Hyperprolactinemic women with normal vitamin D status treated with exogenous vitamin D preparations because of vitamin D deficiency/insufficiency; Group C: Vitamin D-naive hyperprolactinemic women with normal vitamin D status; The data are presented as the mean ± standard deviation; * *p* < 0.05 vs. group A. Abbreviations: BMI—body mass index; HDL—high-density lipoprotein; HOMA1-IR—the homeostatic model assessment 1 of insulin resistance ratio; hsCRP—high-sensitivity C-reactive protein; LDL—low-density lipoprotein; UACR—urinary albumin-to-creatinine ratio.

**Table 4:** Multivariate linear regression analysis for cardiometabolic risk factors.

Variable	Adjusted Partial R^2^ for Prolactin	Adjusted Partial R^2^ for 25-Hydroxyvitamin D
**HOMA1-IR**	0.321 ***	0.278 ***
**Glycated hemoglobin**	0.141 *	0.008 *
**HDL-cholesterol**	0.255 ***	0.134 **
**Triglycerides**	0.243 ***	0.197 ***
**hsCRP**	0.314 ***	0.328 ***
**Fibrinogen**	0.247 ***	0.295 ***
**Homocysteine**	0.312 ***	0.245 ***
**Uric acid**	0.265 **	0.301 ***
**UACR**	0.284 ***	0.253 ***

* *p* < 0.05, ** *p* < 0.01, *** *p* < 0.001. Abbreviations: HDL—high-density lipoprotein; HOMA1-IR—the homeostatic model assessment 1 of insulin resistance ratio; hsCRP—high-sensitivity C-reactive protein; UACR—urinary albumin-to-creatinine ratio.

## Data Availability

The data that support the findings of this study are available from the corresponding author upon reasonable request.

## Abbreviations

BMI—body mass index; HDL—high-density lipoprotein; HOMA1-IR—the homeostatic model assessment 1 of insulin resistance ratio; hsCRP—high-sensitivity C-reactive protein; LDL—low-density lipoprotein; UACR—urinary albumin-to-creatinine ratio.
